# Optimal schedule of adjuvant chemotherapy with S-1 for stage III colon cancer: study protocol for a randomized controlled trial

**DOI:** 10.1186/1745-6215-14-17

**Published:** 2013-01-15

**Authors:** Kenichi Yoshimura, Keisuke Uehara, Yuichiro Tojima, Satoru Kawai, Yasuji Mokuno, Atsuyuki Maeda, Takanori Kyokane, Satoshi Kobayashi, Yuichiro Yoshioka, Masato Nagino

**Affiliations:** 1 Department of Clinical Trial Design & Management, Translational Research Center, Kyoto University Hospital, 54 Shogoin Kawahara, Sakyo, Kyoto, 606-8507, Japan; 2 Division of Surgical Oncology, Department of Surgery, Nagoya University Graduate School of Medicine, 65 Tsurumai, Syowa, Nagoya, Aichi, 466-8550, Japan; 3 Department of Surgery, Chukyo Hospital, 1-1-10 Sanjo, Minami, Nagoya, Aichi, 457-8510, Japan; 4 Department of Surgery, Tsushima City Hospital, 3-73 Tachibana, Tsushima, Aichi, 496-8537, Japan; 5 Department of Surgery, Tokai Municipal Hospital, 1 Marune, Arao, Tokai, Aichi, 476-0003, Japan; 6 Department of Surgery, Ogaki Municipal Hospital, 4-86 Minaminokawa, Ogaki, Gifu, 503-8502, Japan; 7 Department of Surgery, Fukuroi Municipal Hospital, 2515-1 Kuno, Fukuroi, Shizuoka, 437-0061, Japan; 8 Department of Surgery, Toyota Kosei Hospital, 500-1 Ibohara, Jousui, Toyota, Aichi, 470-0343, Japan

**Keywords:** Colon cancer, Stage III, Adjuvant chemotherapy, S-1

## Abstract

**Background:**

Although, in Western countries, oxaliplatin-based regimens have been established as a gold standard treatment for patients with stage III or high risk stage II colon cancer after curative resection, in Japan fluorouracil-based regimens have been widely accepted and recommended in the guidelines for adjuvant settings in patients with stage III colon cancer. S-1, an oral preparation evolved from uracil and tegafur, has equivalent efficacy to uracil and tegafur/leucovorin for treating patients with advanced colorectal cancer and might be a suitable regimen in an adjuvant setting. However, the completion rate of the standard six-week cycle of the S-1 regimen is poor and the establishment of an optimal treatment schedule is critical. Therefore, we will conduct a multicenter randomized phase II trial to compare six-week and three-week cycles to establish the optimal schedule of S-1 adjuvant therapy for patients with stage III colon cancer after curative resection.

**Methods/Design:**

The study is an open-label, multicenter randomized phase II trial. The primary endpoint of this study is three-year disease-free survival rate. Secondary endpoints are the completion rate of the treatment, relative dose intensity, overall survival, disease-free survival, and incidence of adverse events. The sample size was 200, determined with a significance level of 0.20, power of 0.80, and non-inferiority margin of a 10% absolute difference in the primary endpoint.

**Discussion:**

Although S-1 has not been approved yet as a standard treatment of colon cancer in an adjuvant setting, it is a promising option. Moreover, in Japan S-1 is a standard treatment for patients with stage II/III gastric cancer after curative resection and a promising option for patients with colorectal liver metastases in an adjuvant setting. However, a six-week cycle of treatment is not considered to be the best schedule, and some clinicians use a modified schedule, such as a three-week cycle to keep a sufficient dose intensity with few adverse events. Therefore, it will be useful to determine whether a three-week cycle has an equal or greater efficacy and tolerance to side-effects compared with the standard six-week cycle schedule, and thus may be the most suitable treatment schedule for S-1 treatment.

**Trial registration:**

The University Hospital Medical Information Network (UMIN) Clinical Trials Registry UMIN000006750.

## Background

In Japan, colorectal cancer remains the second most common cancer in both genders and the most common cause of cancer mortality in women [1]. The rate of recurrence of stage III colorectal cancer is reportedly 30.8% [2], and adjuvant chemotherapy is important in the treatment of stage III cancer after curative resection. In Western countries, oxaliplatin (Ox)-based regimens in addition to fluorouracil (FU) and leucovorin (LV) (FOLFOX (Ox + FU + LV), FLOX (Ox + FU(, or XELOX (Ox + capecitabine)) have been established as the gold standard adjuvant chemotherapy for stage III colon cancer based on the results of three large randomized controlled phase III studies conducted after 2000 [3-5]. However, favorable results using an FU-based regimen without Ox were reported in a Japanese randomized controlled study (JCOG 0205) [6]. This study compared uracil and tegafur (UFT)/LV with 5-FU/LV as an adjuvant chemotherapy for patients with stage III colon cancer and demonstrated that the three-year disease-free survival (DFS) was 77.8% in the UFT/LV arm and 79.3% in the 5-FU/LV arm, respectively. This result was equal to the experimental arm of the MOSAIC trial using Ox (72.2% in stage III patients only) [3], the NSABP C-07 trial (71.8% including stage II patients) [4] and the XELOXA trial (70.9%) [5]. Therefore, the FU-based regimen has been widely accepted as an adjuvant chemotherapy regimen for stage III colon cancer in Japan, and the Japanese guidelines recommend 5-FU/LV, UFT/LV and capecitabine as equal treatments to FOLFOX [2].

Oral FU is an effective, better tolerated, and convenient chemotherapy regimen compared to Ox-based regimens, as it does not require the use of a central infusion port system and there is less need to visit the outpatient clinic. Therefore, oral FU is a valuable treatment regimen for both patients and medical staffs. The equality of UFT/LV or capecitabine to 5-FU/LV has already been confirmed in large randomized trials [7,8]. S-1 (TS-1, Taiho Pharmaceutical, Tokyo, Japan) is an oral preparation evolved from UFT, which combines tegafur (a prodrug that is converted by cells to fluorouracil), gimeracil (an inhibitor of dihydropyrimidine dehydrogenase, which degrades fluorouracil), and oteracil (which inhibits the phosphorylation of fluorouracil in the gastrointestinal tract, thereby reducing the gastrointestinal toxic effects of fluorouracil) at a molar ratio of 1:0.4:1. Two Japanese phase II trials of S-1 monotherapy for chemotherapy-naïve patients with metastatic colorectal cancer showed that the response rates were 35.5% and 39.5%, similar to the results from the joint study of UFT/LV in the United States and Japan (36.4% in Japan and 34.1% in the United States). In addition, the efficacy of S-1 in an adjuvant setting has been demonstrated in a Japanese phase III trial in patients with stage II or III gastric cancer (ACTS-GC trial) [9]. Currently, two randomized clinical trials are ongoing in Japan to compare the effectiveness of S-1 as an adjuvant chemotherapy with that of UFT/LV or capecitabine in patients with stage III colon cancer [10,11].

The standard schedule of S-1 monotherapy is an oral six-week cycle (four-weeks administration followed by two-weeks rest). However, the completion rate of scheduled treatment in the ACTS-GC trial was reported to be 65.8% [9], which is relatively low, and, moreover, some patients received a modified schedule, such as a three-week cycle (two-weeks administration followed by a one-week rest). Some studies indicated that patients who discontinued treatment early in the adjuvant setting for colon cancer showed poor survival [12,13]. Therefore, it is an urgent necessity to establish the optimal treatment schedule in order to reduce the withdrawal rate of patients receiving S-1 therapy. Only one randomized phase II trial, in which a three-week cycle was compared with a standard six-week cycle has been published. An increase in compliance and a decrease in toxicity was observed in the patients treated with a three-week cycle, and this suggested its use for patients with stage III/IV squamous cell carcinoma of the head and neck after definitive treatment [14]. In this trial, the completion rate along the planned schedule and dose was 40% and 20% in patients treated with a three-week cycle and a six-week cycle and severe adverse grade 3 events were observed in 14% and 27%, respectively.

Based on these findings, we designed the present randomized phase II trial to compare a six-week cycle with a three-week cycle, and to establish the optimal schedule of S-1 adjuvant therapy for patients with stage III colon cancer after curative resection.

## Methods and design

### Purpose

The aim of this study is to determine the optimal schedule of S-1 adjuvant chemotherapy for patients with stage III colon cancer after curative resection.

### Study design

The study is an open-label, multicenter randomized phase II trial, in which participating institutions include thirteen specialized centers in Japan as of August 2012. Participating institutions are listed in Additional file 1: Appendix A.

### Ethical considerations and registration

This study has been conducted in accordance with the Declaration of Helsinki and Ethics Guidelines for Clinical Research by the Ministry of Health, Labor, and Welfare, Japan. Informed consent will be provided for all patients before registration. The trial was approved by the ethics committee of each institution involved in the study. This study was registered at the UMIN Clinical Trial Registry as UMIN000006750 (http://upload.umin.ac.jp/cgi-open-bin/ctr/ctr.cgi?function=brows&action=brows&type=summary&recptno=R000007979&language=E).

### Endpoints

The primary endpoint with respect to efficacy is three-year DFS rate. Secondary endpoints are completion rate of the treatment, relative dose intensity (RDI), overall survival (OS), DFS and incidence of adverse events defined by Common Terminology Criteria for Adverse Events (CTCAE) version 3.0.

### Eligibility criteria

Surgery has to have been performed within eight weeks before induction of the chemotherapy. The primary tumor is staged according to the 7th edition, revised version of the Japanese classification of colorectal carcinoma [15].

### Inclusion criteria

Prior to enrollment in the study, patients must fulfill all of the following criteria: histologically confirmed stage III colon adenocarcinoma (including rectosigmoid cancer); R0 resection for primary tumor; no distant metastases evident on chest, abdominal and pelvic computed tomography (CT); no previous treatment with chemotherapy or radiotherapy for cancer; no current therapeutic treatment with anticoagulants; Eastern Cooperative Oncology Group performance status of 0 to 1; age 20 or older; capable of oral drug intake; and adequate hematological, liver and renal functions as below.

i) White blood cell count of ≥3,500/mm^3^ and <12,000/mm^3^.

ii) Neutrophil count of ≥3,500/ mm^3^.

iii) Hemoglobin of ≥9.0 g/dL.

iv) Platelets of ≥100,000/mm^3^.

v) Total bilirubin of ≤2.0 mg/dL.

vi) AST of ≤100 IU/L and ALT of ≤ 00 IU/L.

vii) Creatinine level of ≤1.2 mg/dL.

viii) Creatinine clearance of ≥60 mL/minute.

Patients have been informed of the investigational nature of the study and have provided their written informed consent before registration.

### Exclusion criteria

Patients may be excluded for any of the following reasons: a synchronous or metachronous active malignancy; serious co-morbidities, such as pulmonary fibrosis or interstitial pneumonia, uncontrollable peptic ulcer disease, poorly controlled diabetes mellitus, severe cardiovascular disease (myocardial infarction, uncontrolled arrhythmia, uncontrolled hypertension) or another serious medical condition; pregnancy or breast-feeding; diarrhea or a peripheral neuropathy greater than Grade 1; or a previous history of severe drug-induced allergy.

### Patient registration and randomization

The participating investigators send an eligibility criteria report to the Data Center and the patients are randomly assigned, in a 1:1 ratio, to either one of the two groups of the S-1 regimen with the different schedules. Computer-generated randomization is performed by minimization methods, stratified by lymph node metastasis (pN1 versus pN2 or pN3) by an institution at a non-profit organization, Medical Support Aichi, Nagoya, Japan. Patient registration began on October 2011 and is to continue for three years.

### Treatment

The patients are randomized to either a six-week cycle (arm A) or a three-week cycle (arm B) of the S-1 regimen.

#### Arm A: six-week cycle

S-1 is given orally at the respective dose for 28 days, followed by a 2-week rest period. Patients are assigned on the basis of body surface area (BSA) to receive one of the following doses twice daily, after breakfast and dinner: BSA < 1.25 m [2], 40 mg; 1.25≤BSA< 1.50 m [2], 50 mg; and BSA ≥ 1.50 m [2], 60 mg.

#### Arm B: three-week cycle

S-1 is given orally at the same dose for 14 days, followed by a 1-week rest period.

If patients had hematologic toxicities of grade 3 or 4 (highest possible grade) or non-hematologic toxicities of grade 2 or higher, their daily dose will be reduced, from 120 mg to 100 mg, 100 mg to 80 mg, or 80 mg to 50 mg. Treatment will be discontinued in patients who had disease recurrence or adverse reactions unable to be controlled by dose modification or temporary withdrawal of S-1. Patients whose toxicities necessitated a rest period of more than two weeks will also be withdrawn from treatment.

### Follow-up

After completion of scheduled treatment, the patients are followed-up until recurrence, other malignancies developed or death is proven, over a period of at least three years. All patients are required to take a blood test, and carcinoembryonic antigen (CEA) and carbohydrate antigen 19–9 (CA 19–9) as tumor marker tests at three-month intervals, and chest, abdominal and pelvic CT at six-month intervals during the first three years. All the patients will be evaluated every three months for the first three years after surgery and every six months for the next two years. The evaluation includes a physical examination, a complete blood count, blood chemical tests, and serum tumor markers. Computed tomography will be performed every six months.

### Sample size determination

This study was based on the hypothesis that a three-week cycle S-1 regimen would be non-inferior to a six-week cycle S-1 for the primary endpoint, with the use of a prespecified non-inferiority margin. The non-inferiority margin was an absolute difference of 10% in the primary endpoint between groups. To test for non-inferiority with a background three-year DFS rate of 75% and an absolute non-inferiority margin of 10%, a total sample size of 200 patients was required for 80% power at a significance level of 20%.

### Statistical analysis

The analysis population for efficacy is the full analysis set. For analysis of the primary endpoint, we estimated the confidence interval (CI) of the hazard ratio using the Cox regression model with a significance level of 20%. Non-inferiority of a three-week cycle S-1 regimen will be claimed if the estimated upper limit of the hazard ratio did not exceed 1.5, which is equivalent to an absolute difference of 10%.

## Discussion

This study is the first randomized trial to investigate the optimal schedule of S-1 therapy for patients with stage III colon cancer. Although S-1 has not been approved yet as a standard treatment in an adjuvant setting, it is a promising option. Moreover, S-1 is a gold standard treatment for patients with stage II/III gastric cancer after curative resection in Japan [9] and is a promising option for patients with colorectal liver metastases in an adjuvant setting. Currently, however, a six-week cycle is not thought to be the best schedule, and some clinicians have selected a modified schedule, such as the three-week cycle as it maintains a sufficient dose intensity with few adverse events. It is extremely valuable to show that a three-week cycle has an equal or greater efficacy and tolerance to side-effects compared with a standard six-week cycle schedule and, therefore, may be the most suitable treatment schedule for the S-1 regimen.

## Trial status

The trial was initiated in October 2011. By 30 July 2012, 38 patients had been registered and randomized.

## Abbreviations

BSA: Body surface area; CA 19–9: Carbohydrate antigen 19–9; CEA: Carcinoembryonic antigen; CI: Confidence interval; CT: Computed tomography; CTCAE: Common terminology criteria for adverse events; DFS: Disease-free survival; FU: Fluorouracil; LV: Leucovorin; OS: Overall survival; Ox: Oxaliplatin; RDI: Relative dose intensity; UFT: Uracil and tegafur.

## Competing interests

The authors declare that they have no competing interests.

## Authors’ contributions

KY and KU equally contributed to develop the study protocols, and wrote most of the final manuscript. KU and MN made substantial contributions to the conception and management in this trial. YT, SK, YM, AM, TK, SK and YY made substantial contributions to the conception and operation of this trial. KY is responsible for statistical design and analysis. All authors were involved in drafting the study protocol and revising it critically. All authors have read and approved the final manuscript.

## Supplementary Material

Additional file 1 **Appendix A.** Participating institutions. Click here for file
