# Supplementary material for: Optimal schedule of adjuvant chemotherapy with S-1 for stage III colon cancer: study protocol for a randomized controlled trial
Source: Trials. 2013 Jan 15;14:17. doi: 10.1186/1745-6215-14-17 (PMC3564899; doi:10.1186/1745-6215-14-17)
Supplement: Additional file 1 — Appendix A. Participating institutions. [file 1745-6215-14-17-S1.pdf]

Appendix A. Participating institution

No	Hospital Name	Principal Investigator
1	Ogaki Municipal Hospital	Atsuyuki Maeda
2	Tsushima City Hospital	Satoru Kawai
3	Chukyo Hospital	Yuichiro Tojima
4	Tokai Municipal Hospital	Yasuji Mokuno
5	Chita City Hospital	Masahiko Asano
6	Nagoya University Hospital	Keisuke Uehara
7	Kasugai Municipal Hospital	Akinori Sasamoto
8	Nagoya Daini Red Cross Hospital	Eiji Sakamoto
9	Toukai Hospital	Hidoeo Yamamoto
10	Toyota Kosei Hospital	Satoshi Kobayashi
11	Anjo Kosei Hospital	Hidenari Goto
12	Toyohashi Municipal Hospital	Kazuhiro Hiramatsu
13	Fukuroi Municipal Hospital	Takanori Kyokane
